# Perioperative extracorporeal membrane oxygenation in pediatric congenital heart disease: Chinese expert consensus

**DOI:** 10.1007/s12519-022-00636-z

**Published:** 2022-11-22

**Authors:** Ru Lin, Wei Wang, Xu Wang, Zhuo-Ming Xu, Jin-Ping Liu, Cheng-Bin Zhou, Xiao-Yang Hong, Xu-Ming Mo, Shan-Shan Shi, Li-Fen Ye, Qiang Shu

**Affiliations:** 1 Department of Extracorporeal Life Support, Heart Institute, National Clinical Research Center for Child Health, Children’s Hospital, Zhejiang University School of Medicine, Hangzhou, China; 2 Department of Thoracic and Cardiovascular Surgery, Shanghai Children’s Medical Center, Shanghai Jiao Tong University School of Medicine, Shanghai, China; 3 Department of Pediatric Intensive Care Unit, National Center for Cardiovascular Disease and Fuwai Hospital, Chinese Academy of Medical Sciences, Peking Union Medical College, Beijing, China; 4 Department of Cardiopulmonary Bypass, National Center for Cardiovascular Disease and Fuwai Hospital, Chinese Academy of Medical Sciences, Peking Union Medical College, Beijing, China; 5 Department of Cardiovascular Surgery, Guangdong Provincial Cardiovascular Institute, Guangdong Provincial People’s Hospital, Guangdong Academy of Medical Sciences, Guangzhou, China; 6 Pediatric Intensive Care Unit, Faculty of Pediatrics, Chinese PLA General Hospital, Beijing, China; 7 Department of Cardiothoracic, Children’s Hospital of Nanjing Medical University, Nanjing, China; 8 Department of CICU, Heart Institute, National Clinical Research Center for Child Health, Children’s Hospital, Zhejiang University School of Medicine, Hangzhou, China; 9 Department of Cardiac Surgery, National Clinical Research Center for Child Health, Children’s Hospital, Zhejiang University School of Medicine, Binsheng Road 3333, Hangzhou 310052, China

**Keywords:** Circulatory support, Congenital heart disease, Extracorporeal membrane oxygenation, Pediatric, Respiratory support

## Abstract

**Background:**

Congenital heart disease (CHD) is one of the main supportive diseases of extracorporeal membrane oxygenation in children. The management of extracorporeal membrane oxygenation (ECMO) for pediatric CHD faces more severe challenges due to the complex anatomical structure of the heart, special pathophysiology, perioperative complications and various concomitant malformations. The survival rate of ECMO for CHD was significantly lower than other classifications of diseases according to the Extracorporeal Life Support Organization database. This expert consensus aims to improve the survival rate and reduce the morbidity of this patient population by standardizing the clinical strategy.

**Methods:**

The editing group of this consensus gathered 11 well-known experts in pediatric cardiac surgery and ECMO field in China to develop clinical recommendations formulated on the basis of existing evidences and expert opinions.

**Results:**

The primary concern of ECMO management in the perioperative period of CHD are patient selection, cannulation strategy, pump flow/ventilator parameters/vasoactive drug dosage setting, anticoagulation management, residual lesion screening, fluid and wound management and weaning or transition strategy. Prevention and treatment of complications of bleeding, thromboembolism and brain injury are emphatically discussed here. Special conditions of ECMO management related to the cardiovascular anatomy, haemodynamics and the surgical procedures of common complex CHD should be considered.

**Conclusions:**

The consensus could provide a reference for patient selection, management and risk identification of perioperative ECMO in children with CHD.

Video abstract (MP4 104726 kb)

**Supplementary Information:**

The online version contains supplementary material available at 10.1007/s12519-022-00636-z.

## Introduction

Until the 1970s, extracorporeal membrane oxygenation (ECMO) was reported to be successfully used in a neonate with acute respiratory failure [1]**.** Since the 1980s, the number of cardiac ECMO cases has increased dramatically, though neonatal respiratory failure remains to be the most common indication for ECMO support [2]. ECMO is used for respiratory support, circulatory support and cardiopulmonary resuscitation (CPR) in pediatrics**.** The most common neonatal diagnosis requiring respiratory ECLS (extracorporeal life support) was congenital diaphragmatic hernia (32%), followed by meconium aspiration syndrome (24%) and persistent pulmonary hypertension (21%) [3]. The most common disease requiring circulatory ECLS is congenital heart disease (CHD), 80% in newborns and 52% in children [3].

Veno-arterial ECMO (VA ECMO) is the main support mode for CHD. According to the data from Extracorporeal Life Support Organization (ELSO) registry, a total of 19,629 cases with CHD from 350 international centers worldwide from 1990 to 2019 have been supported by ECMO [4]. Among them, the most common condition was hypoplastic left heart syndrome (HLHS) in neonates, whereas in children conditions requiring complex biventricular repair (such as tetralogy of Fallot, double outlet of right ventricle, Ebstein's anomaly of the tricuspid valve) were the most common [4]. The median ECMO running duration for of pediatric cardiac diseases was 6–7 days, and the survival rate was 46%–55%, which was significantly lower than other classifications of diseases [4].

In China, ECMO was reported to be successfully used in a neonate with CHD in 2008 [5]. A total of  10,946 patients with CHD underwent cardiopumonary bypass from January 2017 to June 2020, ECMO cases accounted for 1.21%  and a survival rate was 47% [6]**. **In 2021, ECMO was used for respiratory support in 126 children and for circulatory support in 422 children with a survival rate of 59.5% [7]. A total of 127 children underwent external cardiopulmonary resuscitation (ECRP) support with a survival rate 39.4%, and among them 10 were newborns with a survival rate of 8.3%. A total of 65 newborns with CHD underwent ECMO with a survival rate of 43.1% [7].

ECMO support after arterial switch operations (ASOs) accounted for 21.2% of total ECMO cases with CHD [8]. Children with CHD have more challenges due to complex heart anatomical structure, special pathophysiology, perioperative complications and various concomitant malformations. This consensus focused on the technical approach of ECMO support in pediatric CHD. Recommendations on patient selection, management and risk identification of perioperative ECMO support in pediatric CHD were provided.

## Patient selection criteria

### Indications

Most neonatal cardiac ECMO occurs during the perioperative period and particularly the post-procedure period [9]. Perioperative ECMO in children with CHD is used to stabilize and recover the respiratory and circulatory function. ECMO may also be used for recovery from the primary disease and providing time for follow-up diagnosis and treatment and for awaiting other therapeutic modalities, such as ventricular-assist device (VAD) or transplantation in the following situations: (1) pre-procedural patients with severe hypoxemia and low cardiac output syndrome (LCOS): patients with total anomalous pulmonary venous connection and transposition of the great arteries (TGA) may present with severe hypoxia, acidosis and LCOS due to insufficient oxygenation of blood and persistent pulmonary hypertension; (2) failure to wean off cardiopulmonary bypass (CPB): ECMO support may be required for patients who fail to wean off CPB after cardiac surgery for improper myocardial protection, myocardial ischemia injury or residual anatomical deformity; (3) severe postoperative LCOS: LCOS occurs due to myocardial damage or stunning, coronary ischemia, malignant arrhythmia, residual lesions and sudden obstruction of the postoperative systemic-pulmonary shunt; (4) cardiac arrest due to a variant of causes.

ECMO should be initiated as early as possible if with the following indications [10–16]: (1) cardiac index < 2 L/m^2^/minute; (2) persistent tissue hypoperfusion: blood pH < 7.15, BE < -5 mmol/L, lactate > 7.3 mmol/L, urine output < 1 mL/kg/hour, capillary refill time > 3 seconds, central venous oxygen saturation (S_V_O_2_) < 60% or arteriovenous oxygen saturation difference (AVO_2_) > 30% for cyanotic CHDs; (3) persistent hypotension: the blood pressure is two standard deviations lower than that of normal blood pressure at the same age, such as for systolic blood pressure < 50 mmHg (neonates), < 60 mmHg (infants) and  < 70 mmHg (children); (4) patients presenting with low blood pressure under two or more high-dose inotropes and/or vasopressors, such as epinephrine > 0.3 µg/kg/minute, dopamine > 10 µg/kg/minute, etc., or vasoactive–inotropic score is over 20 for two or more times and keeps increasing; (5) severe respiratory failure: severe hypoxia or respiratory acidosis persists even after conventional aggressive treatment, such as pH < 7.1, partial pressure of oxygen/fraction of inspired oxygen (PaO_2_/FiO_2_) < 60–80 mmHg or oxygenation index > 40, lasting 3–6 hours; (6) malignant arrhythmias: ECMO should be considered when severe arrhythmias, such as ventricular fibrillation, cardiac arrest or pulseless electrical activity and short bursts of ventricular tachycardia occur repeatedly and cannot be terminated by antiarrhythmic drugs, inotropes or temporary cardiac pacemakers. Consideration for early initiation of ECMO is important as delayed initiation (beyond 6 hours of cardiogenic shock state) is associated with worse outcomes.

### Contraindications

ECMO should not be used under the following conditions [10]: (1) prolonged cardiogenic shock state (more than 6 hours) complicated with irreversible multiple organ failure; (2) premature or low birth weight neonates (< 34 weeks of gestational age or weight < 2.0 kg); (3) severe chromosomal abnormalities; (4) irreversible brain injury or intracranial hemorrhage (grade III or IV intraventricular hemorrhage); (5) uncontrolled bleeding (unless ECMO cannulation can help to control bleeding).

## Cannulation strategy

VA ECMO was the most commonly used mode for most congenital heart diseases perioperatively. Cannulation site and strategy were determined by the patient’s body size, underlying cardiac anatomy and different surgical procedures for CHD. The commonly used cannulation strategy is listed in Table 1. Central cannulation is commonly used in failure to wean from CPB or in the presence of recent sternotomy (less than 10–14 days). The right neck or femoral vessels can be considered as the peripheral cannulation site. Most commonly, the right internal jugular vein and carotid artery configuration is used in younger patients (weight < 30 kg), while the femoral vein and femoral artery configuration is used in older patients (weight > 30 kg) [10]. Each ECMO center selects a peripheral cannulation site based on technique preference with reference to the patient’s weight range.

**Table 1:** Cannulation strategy in children with cardiac disease

Anatomy or surgical palliation	Central cannulation		Peripheral cannulation		Additional strategies
	Venous access	Arterial access	Venous access	Arterial access	
Two ventricles					
Biventricular circulation or structurally normal heart	Right atrium	Aorta	Internal jugular or femoral	Common carotid or femoral	Left atrial decompression may need to be considered
Single ventricle					
Shunted or RV-PA conduit physiology (stage 1)	Common atrium	Aorta	Internal jugular	Common carotid	Peripheral: neck access due to patient size Care: cannula position with respect to shunt—may result in overcirculation to lungs or shunt occlusion
Superior cavopulmonary anastomosis (stage 2)	SVC or common atrium	Aorta	Internal jugular or femoral	Common carotid	If femoral approach only used, passive venous return must flow through lungs—ventilation must be optimized Additional venous cannula may be required
Total cavopulmonary anastamosis (Fontan, stage 3)	Fontan baffle or common atrium	Aorta	Internal jugular or femoral	Common carotid or femoral	Additional venous cannula often required

*RV-PA* right ventricle-to-pulmonary artery, *SVC* superior vena cava [10]

Peripheral vessel cannulation requires usually open surgical access [10]. The tip of the arterial cannula is located at the junction of the innominate artery and the aortic arch, preventing access to the ascending aorta. The tip of the venous cannula is located at the right atrium, preventing access to the right ventricle or hepatic vein. Single-ventricle patients with ductal- or shunt-dependent pulmonary blood flow may require excessive ECMO flow rates to accommodate the runoff into the pulmonary vascular bed and to provide adequate systemic tissue oxygen delivery. When a high volume of flow can be difficult to achieve from peripheral cannulation, central cannulation may be necessary [2]. When patients following the Glenn or Fontan procedure require VA ECMO, venous drainage cannulas may be possible in both the superior and inferior vena cava to achieve adequate vein drainage [17].

## ECMO pump flow, ventilator parameters and vasoactive drug dosage setting

During ECMO, mixed venous oxygen saturation (S_V_O_2_) should be continuously monitored. The cardiac index should be maintained at 2.5–3 L/minute/m^2^ and the ratio of oxygen delivery (DO_2_) to oxygen consumption (VO_2_) should be set at least > 3:1 by adjusting pump flow and hemoglobin level [10]. Reasonable ventilator parameters and vasoactive drugs help to restore cardiac function and maintain perfusion of the organs and peripheral microcirculation (Table 2).

**Table 2:** Indications of ﻿vasoactive drugs for pediatric cardiac

Vasoactive drugs	Indication, benefits, and specific risks	Medication	Starting dose range
Inotrope	Enhancement of contractility in a patient with severe cardiac dysfunction to facilitate aortic valve opening, and prevent stasis of blood in the systemic ventricle and aortic root To optimize blood pressure and end-organ perfusion Not facilitate myocardial rest	Epinephrine Dobutamine	0.02–0.05 μg/kg/min 5 μg/kg/min
Vasopressor	Peripheral vasoconstriction is indicated in a patient on VA ECMO for distributive shock, on maximal circuit blood flow with inadequate cardiac output to optimize blood pressure and end-organ perfusion	Norepinephrine Vasopressin	0.02–0.05 μg/kg/min 0.01–0.06 IU/kg/h
Vasodilator	Peripheral vasodilation will reduce systemic afterload improving circuit blood flow and systemic perfusion as well as decreasing left ventricle afterload, promoting ejection	Sodium nitroprusside Milrinone Nitroglycerin	0.5-3 μg/kg/min 0.25–1.0 μg/kg/min 0.3–0.6 μg/kg/min

*VA ECMO* veno-arterial extracorporeal membrane oxygenation [4]

During the initial period, higher blood flow is required for early oxygen debt repayment, typically 100–150 mL/kg/minute in neonates and 80–120 mL/kg/minute in children [10, 18]. Pulmonary blood flow is reduced during VA ECMO, so the ventilation volume should be reduced proportionally [19]. Protective lung ventilation strategies with low parameter settings, including setting positive end-expiratory pressure (PEEP) 8–10 cm H_2_O and tidal volume < 6–8 mL/kg, peak inspiratory pressure < 18–20 cm H_2_O and frequency 10–15 times/minute can be utilized to reduce pulmonary complications [13, 19, 20]. High PEEP increases intra-thoracic pressure, pulmonary vascular resistance and right ventricular afterload, which adversely affect children with predominant right heart failure [21]. Conversely, children with predominant left heart failure often benefit from a high PEEP [19]. High pump flow will increase left ventricular afterload and myocardial performance after ECMO implantation; vasopressors should be reduced or discontinued as soon as possible and vasodilators administered to reduce afterload, to promote recovery of myocardium and microcirculation perfusion and to avoid important organ and extremity complications. Maintenance of normal rhythm with drugs or pacemakers improves ventricular emptying. In children with severe right heart failure and pulmonary hypertension, inhaled nitric oxide and/or targeted pulmonary arterial vasodilators can be used to reduce right heart afterload [13]. It is necessary to precisely regulate fluid balance, ventilator parameters and ECMO pump flow after ASO and anomalous left coronary artery origin from pulmonary artery (ALCAPA) repair. The regulation strategy should be tailored to improve the left ventricle adaptability to preload and afterload and avoid left ventricular distension. For children with HLHS, low pulmonary vascular resistance may lead to reduced systemic circulation and hypotension, whereas excessive pulmonary vascular resistance may lead to hypoxia. Children with B-T shunts or aortopulmonary collateral arteries require higher blood flow (150–200 mL/kg/minute) to accommodate the runoff into the pulmonary vascular bed [4, 22]. Additionally, when the flow of the shunt or the collateral artery is too great, the shunt or the collateral artery needs to be partially clamped or blocked.

## Anticoagulation management

Bleeding is a common complication of ECMO for post-cardiac surgery patients, most frequently in central cannulation [23].The main causes of bleeding are the immature coagulation function of neonates and infants, the dilution of coagulation factors after connection to the ECMO circuit, the large open wound and the coagulopathy usually associated with a long duration of CPB. The main methods to reduce bleeding include reversing partial heparin with limited protamine administration [24], strict surgical hemostasis, and correcting coagulopathy and thrombocytopenia by infusing fibrinogen, fresh frozen plasma and platelets. If excessive bleeding occurs, particularly in post-cardiotomy patients, delayed infusion of unfractionated heparin (UFH) persistently for 4–6 hours is required. Under some circumstances where bleeding is difficult to control, UFH may be held up for 12 hours or longer until bleeding is controlled [25]. Delayed initiation of anticoagulation will increase the risk of circuit clots, which will increase the consumption of fibrinogen and platelet. UFH is currently the most commonly used anticoagulant for ECMO. The effectiveness of heparin can usually be monitored using activated clotting time (ACT) and activated partial thromboplastin time (APTT) combined with anti-Xa, thromboelastometry (ROTEM) and thromboelastography (TEG) [17]. ROTEM or TEG is currently recommended to guide the administration of blood products and coagulation factors in the presence of bleeding [26].

A bolus dose of UFH ranging from 50 to100 units/kg is given after the exposure of the vessels and before insertion of the cannulas for ECMO. Patients with severe coagulopathy, or active bleeding can receive a UFH bolus at the lower end of this range. Nevertheless, in the immediate postoperative transferring from CPB, bolus dosing of UFH may not be necessary [27]. UFH can be commenced when ACT reaches 300 seconds [18] and chest tube drainage is < 3 mL/kg/hour for 2 hours [28]. Some ECMO centers set maintenance dose of UFH infusion rate ranging from a minimum of 10–15 units/kg/hour to a maximum of 40–60 units/kg/hour [18]. The targeted ACT 180–220 seconds and APTT 1.5 and 2.5 times normal are needed. Targeted ACT value can be lowered to 160–180 seconds when there is a tendency to bleed. Blood production can be given to maintain platelet counts ≥ 100 × 10^9^/L (bleeding patient) or  ≥ 50–100 × 10 ^9^/L (nonbleeding patient), fibrinogen > 1.5 g/L (bleeding patient or before surgical intervention) or  > 1 g/L (nonbleeding patient). If a maximum dose of UFH cannot achieve the targeted ACT value, fresh frozen plasma could be infused to maintain antithrombin activity at > 50%–80% or 0.5–0.8 U/mL [25].

Direct thrombin inhibitors (DTIs) have been used in both adult and pediatric patients with heparin-induced thrombocytopenia (HIT), heparin resistance and non-HIT thrombocytopenia [29, 30]. The two DTIs most commonly used in ECMO are bivalirudin and argatroban. APTT is currently the standard test for monitoring DTIs (target 50–60 seconds), and the reported maintenance dose of bivalirudin and argatroban ranges from 0.045 to 0.48 mg/kg/hour and 0.1 to 0.7 μg/kg/minute, respectively [18]. Currently, experiences of using DTIs are lacking in pediatric ECMO in China, and clinicians should be aware of the safety and efficacy of DTIs.

## Left ventricle decompression

When severe left ventricle (LV) dysfunction is supported with VA ECMO, continuous flow increases the pressure of the aortic root and LV afterload [31, 32]. Poor LV decompression may result in a persistently closed aortic valve, dilatation of the left heart, increased LV end-diastolic pressure, reduced sub-endothelial perfusion causing myocardial ischemia and poor LV function recovery, and a risk of LV stasis and consequent clot formation [33, 34]. It is important to recognize poor LV decompression early and to perform effective LV decompression.

After cardiac tamponade was excluded, the implications of poor LV decompression were reduced arterial pulse pressure (< 10 mmHg), increased pulmonary capillary wedge pressure (PCWP) or left atrium (LA) pressure (> 20 mmHg), pulmonary edema, closed aortic valve, increased LA or LV end-diastolic diameter and aggravated mitral valve regurgitation [35].

Conservative decompression was the first-line strategy: (1) titrate the ECMO flow [35] and use a vasodilator to decrease LV afterload; (2) increase inotrope to improve LV output; and (3) increase PEEP to decrease LV preload [35]. If conservative methods do not work effectively, invasive strategies can be utilized: (1) LA venting via the right-upper pulmonary vein or left atrial appendage (most commonly in post-cardiac surgery); (2) balloon atrial septostomy or blade atrial septostomy to allow left-to-right shunting [35, 36]; (3) a venting cannula via the LV apex or pulmonary artery [37, 38]; and (4) intra-aortic balloon pump or a percutaneous LV-to-aorta ventricular-assist device, such as impella, could be used in adolescents or young adults [2, 35, 39].

## Residual lesions

Postoperative residual lesions of CHD can significantly increase the risk of postoperative complications and mortality rate [40]. Therefore, transesophageal echocardiography during the operation to detect residual lesions and timely intervention are conducive to the smooth withdrawal of CPB [41–43]. If there is no sign of myocardial function recovery after 48–72 hours of ECMO support, it is necessary to positively identify residual anatomical malformation. Identifying residual lesions and intervening during the first three days of ECMO support can significantly shorten support duration and improve the survival rate of patients [40, 41].

Common residual lesions include residual ventricular septal defects, stenosis and dysplasia of the pulmonary artery branch, stenosis of the left and right ventricular outflow tracts, pulmonary vein stenosis, B-T/Sano duct stenosis or too great, severe valvular regurgitation, bulky pulmonary collateral and coronary artery disease [41, 42]. In the early stage of support, residual lesion can present as ventricular dilatation and dyskinesia, insufficient systemic perfusion caused by excessive shunt, suboptimal blood lactate decrease, pulmonary hemorrhage, aberrant increase in mixed SvO_2_, etc. Children with a single ventricle may have insidious manifestations and may not present a significant increase in the left atrial or central venous pressure until the auxiliary flow is reduced to 30%–40%.

The first option for examination to identify residual anatomical malformation is ultrasound [41, 42]. When processing the examination, we can reduce the flow of ECMO or clamp the circuit temporarily to evaluate the cardiac function more accurately and identify residual lesions. Many factors, such as wound dressings, opened sternum, various cannulations and inappropriate size of ultrasound probe, can affect the acquisition of standard ultrasound views. If necessary, further examinations, such as cardiac catheterization or computed tomography angiography (CTA), can be recommended [43–45].

## Fluid management and renal replacement therapy

Children with VA ECMO during the perioperative period of CHD need appropriate fluid to maintain cardiac output and ECMO flow. Dynamic monitoring of central venous pressure (CVP), PCWP, pre-pump pressure of circuit and other comprehensive evaluations of volume status are crucial. During the early period of ECMO support, bleeding, insensible fluid loss or capillary leakage syndrome may be attributed to insufficient ECMO flow. When the pre-pump pressure of ECMO becomes more negative (below -40 to -30 mmHg), the venous line vibrates or the inferior vena cava collapses [46] and colloids and low dose of vasoactive drugs can be given firstly to maintain the intravascular volume. Once the circulation of children is stable and capillary leakage is improved, fluid intake should be limited and negative fluid balance is warranted to avoid fluid overload. Renal replacement therapy should be actively applied in case of intravascular fluid overload, oliguria or refractory to diuretics and albumin, elevated creatinine and electrolyte disorder [10, 47].

About 49%–59% of children on ECMO with CHD developing acute kidney injury (AKI) and fluid overload require renal replacement therapy (RRT) [48, 49]. Low cardiac output, hypotension, venous congestion, non-pulsatile perfusion, inflammatory reaction and hemolysis may cause AKI [50] and fluid overload, prolonging ECMO supporting time and increasing mortality [51–54]. The commonly used RRT models in children are continuous renal replacement therapy (CRRT) [50] and peritoneal dialysis (PD). Although some researchers reported no improvements in the survival rate by utilizing ECMO with CRRT and no high-level evidence or guidelines support routine usage of CRRT during ECMO, some researchers discovered that the combination of ECMO and CRRT may be effective [51, 55, 56].

**Fig. 1:**
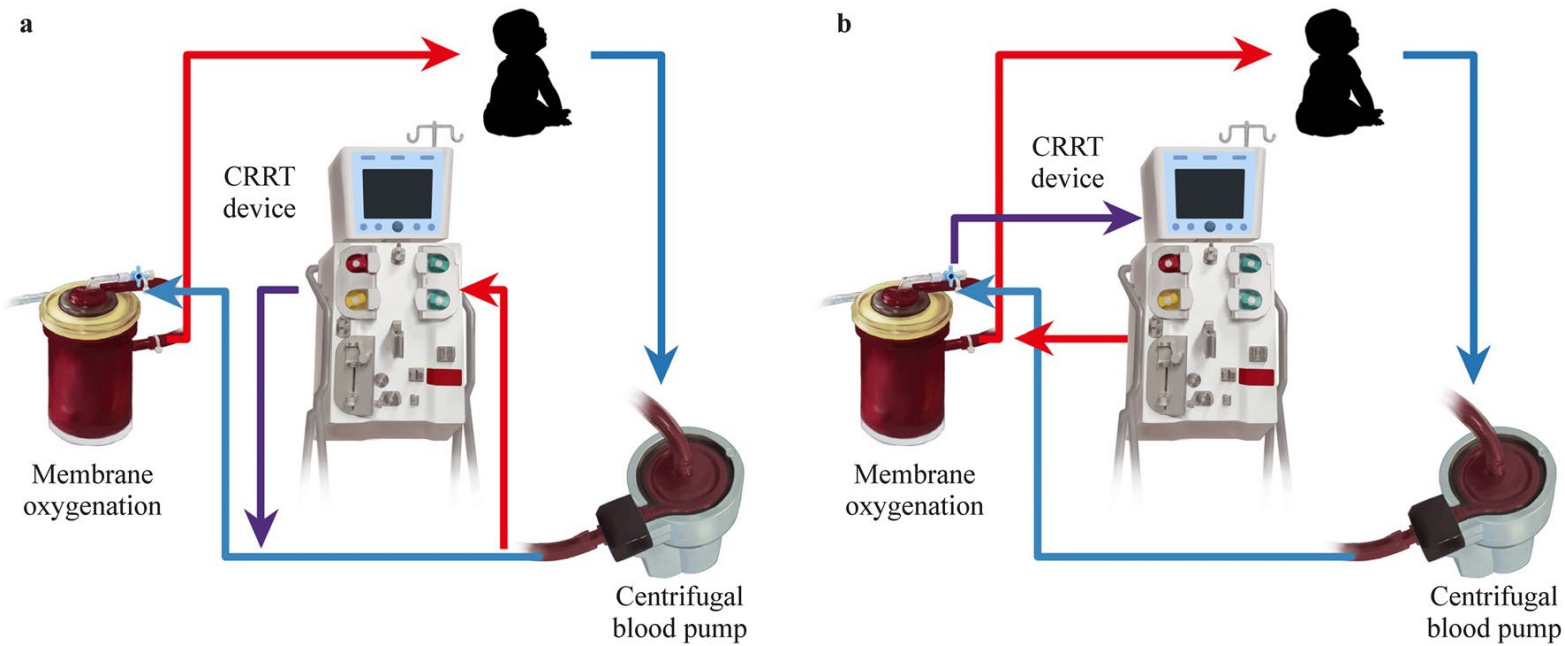
The connection of ECMO with continuous renal replacement therapy (CRRT)

To avoid serious air embolism, we recommend the input and output interfaces of CRRT be connected to the post-pump part of the ECMO circuit (Fig. 1). Parallel connection with the CRRT circuit will increase the turbulence and coagulation risk of ECMO circuit interface, with the need for close monitoring for hemolysis, bleeding and coagulation [50]. If the patient has undergone CRRT before ECMO support, the original CRRT catheter is recommended to be continued. According to the KDIGO (Kidney Disease: Improving Global Outcomes) guidelines [57], for those who have already received systemic anticoagulation with UFH, other anticoagulants are generally not added. Some researchers [58] have proposed regional citrate anticoagulation to prolong the life span of the CRRT filter. Each center can make systematic assessment and decide the individual connection mode and anticoagulation schemes.

The safety and effect of peritoneal dialysis during ECMO in children with CHD should be affirmed [59–63]. Because anticoagulation increases the risk of bleeding during PD tube placement, the patient’s coagulation status should be evaluated before placement to prevent bleeding. Ultrasound guidance can avoid the risk of abdominal complications (such as intestinal perforation, parenchymal organ puncture injury).

## Wound care

Central cannulation generally requires delayed sternal closure. The sternal skin should be sutured as much as possible or covered with sterile dressings [64]. Strict aseptic techniques, keeping the wound surface clean and dry, assessing the hemorrhage and exudation, routine cleaning and sterilizing the skin around the cannula and incision, and pericardial mediastinal irrigation are essential to prevent infections. Topical sealants, such as gelatin sponges or dry gauze strips, can be used to control oozing and bleeding [65]. When chest drainage increases or stops with increased tension of the dressing, accompanied by decreasing blood pressure and pulse pressure, lower cardiac sound and unstable pump flow [66], pericardial tamponade should be considered. Emergency thoracotomy to remove the accumulated blood is recommended.

## Weaning and transition

When cardiac function recovers after 5–7 days of ECMO support, weaning can be considered. If there is no sign of improvement in cardiac function for longer than two weeks, transition to an intermediate- or long-term mechanical cardiac support device or heart transplant should be considered [67]. If irreversible multiple organ failure occurs, ECMO withdrawal should be considered. Before VA ECMO weaning, it is necessary to repeatedly evaluate cardiopulmonary function, residual lesions and adaptive changes in the cardiac structure. Reducing the pump flow and temporarily clamping the circuit are needed for further evaluation.

When the pump flow is gradually reduced to less than 30% of the full flow for 6–24 hours, weaning can be attempted if the following conditions are met [10, 68–70]: (1) with low levels of vasoactive drugs (epinephrine ≤ 0.02–0.05 μg/kg/minute, dopamine/dobutamine 3–5 μg/kg/minute), arterial pressure within the normal values and pulse pressure difference greater than 20 mmHg, CVP > 5 mmHg [10]; (2) left ventricular ejection fraction > 25%, left and right ventricular motion coordination and aortic valve velocity time integral ≥ 10 cm[69]; (3) with the ventilator parameters as follows: oxygen concentration ≤ 60%, tidal volume 6–8 mL/kg, peak inspiratory pressure < 30 cmH_2_O, PEEP 5–15 cmH_2_O and PaO_2_/FiO_2_ > 200 mmHg after biventricular correction, PCO_2_ ≤ 45 mmHg, pH > 7.3, SvO_2_ ≥ 65%, blood lactate, electrolyte and hematocrit (HCT) values within the normal range; (4) in patients with a single ventricle, it is necessary to adjust the balance of systemic/pulmonary blood flow according to the cardiac surgical procedures and the level of peripheral blood oxygen saturation (SpO_2_). The oxygenation index and mechanical ventilation parameters should meet the targets and all conditions remain stable.

First-aid medicines, equipment and blood availability should be accessible to address the possible risks during weaning. The trial off VA ECMO for CHD is similar to the ELSO guideline [10, 68, 71, 72]. The LV decompression cannula should be removed first. The sternal incision could be closed at the same time as decannulation if hemodynamics is stable. If the carotid artery is difficult to repair, ligation should be considered. It is still necessary to evaluate cardiopulmonary function after weaning off. If cardiopulmonary function is unstable, ECMO should be implanted once again.

## Main complications

### Bleeding and thromboembolism

Bleeding after cardiac surgery could be caused by surgical factors or coagulation disorder. The optimal treatment depends on the cause, amount and site of bleeding. For surgical bleeding, operative hemostasis should be performed. For light bleeding other than the brain, the anticoagulation level can be adjusted downward. In cases of intracranial hemorrhage and/or massive bleeding, anticoagulants should be suspended until the bleeding is controlled. Hemostatic and blood products should be given as needed. For refractory bleeding, anti-fibrinolytic therapy (e.g., tranexamic acid) should be initiated if necessary.

ECMO-associated thrombosis could occur both in the ECMO circuit and vessels. Coagulation disorders after prolonged CPB, high-dose vasopressor infusion, delayed initiation of heparin and excessive infusion of coagulation substrate increase the risk of thrombosis. An individual anticoagulation strategy, careful surgical hemostasis, stable internal environment, reasonable infusion of coagulation substrate, appropriate vasopressor usage, effective left heart decompression [73] and reduction of ventricular blood stasis could contribute to reduce the risk of thrombosis. Small thrombi in the ECMO circuit should be observed closely by improving the anticoagulation level. In case of life-threatening thrombotic events, emergency treatment of surgical thrombectomy [74] and ECMO circuit replacements should actively be taken into consideration.

### Brain injury

The risk factors for ECMO-related brain injury include younger age (gestational age < 34 weeks), lower body weight (< 3 kg), exposure to CPR, acidosis, surgical factors, thromboembolism, systemic anticoagulation and cerebral hypoperfusion [76]. Routine neurological assessment and multimodal neurological function monitoring [76], such as transcranial ultrasound, continuous near-infrared spectral oxygen saturation, electroencephalography and bispectral index, should be performed to monitor possible cerebral dysfunction. A CT scan is recommended to evaluate the possibility of acute brain injury in cases with clinical signs. In cases with cerebral edema, slightly lowering the circuit temperature, local sub-hypothermia therapy, glucocorticoids and dehydrating agents should be used to control the intracranial pressure. It is necessary to monitor CO_2_ levels to avoid the effects of hyperventilation on cerebral vessels. In patients with mild intracranial hemorrhage, close monitoring of neurological function and optimization anticoagulant strategy are required. ECMO discontinuation should be considered in cases of severe intracranial hemorrhage with an expected poor prognosis.

### Extracorporeal cardiopulmonary resuscitation

ECPR is the rapid deployment of VA ECMO to provide reperfusion with oxygenation and cardiac support when conventional cardiopulmonary resuscitation (CPR) fails to restore sustained spontaneous circulation [77]. Owing to the anatomical and physiological characteristics of CHD, low cardiac output, poor oxygenation and insufficient cerebral perfusion are prone to occur during chest compression. Therefore, when CPR fails to restore spontaneous circulation within 15 minutes, the ECPR procedure should be started immediately [70, 77]. Timely application of ECPR can reduce organ damage and improve the survival of children with CHD in the hospital [78–81].

According to the anatomical characteristics of CHD and the speed of rescue, the ECPR cannulation strategy is formulated, central cannulation can be performed in the early stage within the postoperative 14 days, and cervical or femoral arteriovenous cannulation can be performed in children before surgery or during the late postoperative period [70, 82].

One of the key factors for the success of ECPR is the full effectiveness of CRP and the rapid implementation of ECMO [83, 84]. After ECMO initiation, high pump flow is given to improve the perfusion, oxygenation and inner environment of organs as soon as possible to create the conditions for subsequent diagnosis and treatment. Although effects of mild hypothermia brain protection is controversial, targeted management of mild hypothermia (33–34 °C) for 24–48 hours may be considered in children at risk of serious neurological complications [85]. Carrying out imaging examinations, identifying the primary cause of cardiac arrest and implementing timely intervention or surgical re-intervention will help to improve the prognosis [82, 86, 87].

## Special considerations for complicated CHD

### Hypoplastic left heart syndrome

ECMO management differences after Norwood surgery for HLHS is related to the procedures of Sano or B-T shunt. After Sano shunt, pulmonary vessels only receive blood supply in ventricular systolic stage, whereas after B-T shunt, pulmonary vessels receive continuous blood supply in the systolic and diastolic stages, and the latter is prone to resulting in a greater risk of coronary insufficiency. To provide sufficient systemic and coronary perfusion during ECMO running, in addition to strengthening cardiac function and maintaining higher HCT level, excessive reduction in pulmonary vascular resistance should be avoided by adjustment of medicine and ventilator setting.

### Single-ventricular, bidirectional Glenn and Fontan circulation

For uncorrected or stage 1 single ventricle, when patent ductus arteriosus (PDA) (or B-T shunt) closure and/or restricted atrial septal defect (ASD) occurs, insufficient pulmonary and systemic blood mixing leads to severe hypoxemia, elevated pulmonary arterial and venous pressure and finally to circulatory failure. Under these conditions, ECMO support should be considered. A high ECMO flow rate (150–200 mL/kg/minute) is usually required to compensate for the flow shunt into the pulmonary circulation [17]. Emergency atrial septostomy or corresponding surgery is required when hemodynamics is stable.

Bidirectional Glenn and Fontan circulations rely on passive low pulmonary resistance. When central cannulation is adopted after early surgery, superior vena cava cannulation should be established first to achieve venous drainage and reduced intracranial pressure to decrease intracranial bleeding risks. If venous drainage is insufficient, inferior vena cava cannulation should be added. Excessive ventilation causing low CO_2_ blood levels perhaps might lead to insufficient cerebral blood flow, further resulting in insufficient venous drainage [10, 17].

When higher pump flow is provided, maintaining pulsatile blood flow by a certain degree of ventricle filling and output is needed to avoid blood stasis in Fontan graft [17]. In patients with chronic cardiac insufficiency, increased afterload induced by higher pump flow may not be conducive to myocardial recovery and ECMO withdrawal [17]. Due to the lack of a sub-pulmonic pumping/capacitance chamber, compressions and recoil by chest outside massage often result in blood moving back through the venous chamber rather than antegrade through the lungs and into the systemic ventricle for coronary artery and cerebral perfusion. Once cardiac arrest happens in patients after Fontan procedure, ECMO should be established as soon as possible [17].

Various causes of bidirectional Glenn and Fontan circulation failure should be investigated, such as arrhythmia, anatomic obstruction to flow, pulmonary vascular remodeling, atrioventricular valve dysfunction, univentricular diastolic dysfunction and chronic under filling and/or univentricular systolic dysfunction. As one might imagine, the outcome of extracorporeal support largely depends upon the underlying physiology and mechanism for “Fontan failure” [17].

ECMO is effective for early postoperative support of Fontan circulation failure. However, patients with late-phase failure usually present with extreme end-organ failure, such as protein losing enteropathy, plastic bronchitis, cirrhosis, or renal failure. The middle-to-late phase failure patients are appropriate transplant candidates and are better suited for transitioning to more durable mechanical support (ventricular-assist device) via ECMO [17].

### Transposition of the great arteries with intact ventricular septum

Pulmonary hypertension is uncommon in patients with TGA/IVS). The incidence of TGA/IVS with pulmonary hypertension was 1%–12% [88, 89]. Increased pulmonary vascular resistance leads to reduced pulmonary blood flow and atrial blood does not mix effectively. Coupled with PDA right-to-left shunt, effective pulmonary blood flow is further lost. The VV or VA ECMO mode can be adopted. The time of ECMO supporting should not be too long and both pulmonary hypertension and left ventricular “deconditioning” resulting from preoperative use of ECMO are responsible for left ventricular mass decaying and functional degeneration [89, 90]. ASO should be performed as soon as possible when the condition is stable. When the uncorrected TGA/IVS with restricted ASD and without pulmonary hypertension develops severe hypoxemia, it is not suitable to establish the ECMO, but emergency ASO or atrial septostomy should be given priority because the risk of pulmonary hemorrhage and left heart dramatic expansion owing to restricted ASD and excessive pulmonary blood coming from PDA is dramatically increased.

### Other conditions

#### Aortic regurgitation

VA ECMO blood flow may aggravate aortic regurgitation and increase the risk of left ventricular afterload and left ventricular dramatic expansion, so aortic regurgitation should be assessed carefully before ECMO implantation. If it is found after ECMO implantation, early left ventricular decompression or surgical correction is required [10].

#### Aortic arch separation

Careful attention to the anatomy of the head and neck vessels (i.e., location of interruption of arch) is required before ECMO cannulation to ensure brain perfusion with oxygenated ECMO flow [10].

#### Anomalous left coronary artery origin from pulmonary artery

Due to secondary severe myocardial ischemia, most of the ALCAPA patients had severe cardiac insufficiency, with high incidence of perioperative adverse events [91, 92]. Planned application of ECMO could be carried out for children with severe LV insufficiency. If cardiac function cannot be improved within a short period postoperation, central cannulation can be changed to peripheral cannulation or converted to VAD, which could be beneficial for chest closure, prolonging ventricularassisting time and reducing infection risks.

## Conclusions

This consensus provided a comprehensive clinical and technical approach for ECMO support in CHD in children. Indications and contraindications should be evaluated carefully before ECMO application. The cannulation strategy is determined based on the patient’s body size, cardiac anatomy and different surgical procedures of CHD. ECMO pump flow, ventilator parameters and vasoactive drugs should be adjusted according to different ECMO support stages and goals as well as special characteristics of cardiac function, procedures and hemodynamics, to maintain the balance of DO_2_ and VO_2_ and to promote the recovery of cardiac function and organ protection. The anticoagulation strategy should be tailored by considering the unique bleeding and clotting risks. Left ventricular decompression and pericardial tamponade should be monitored carefully in the early stage of ECMO. Residual lesions, volume overload, bleeding and brain complication should be identified and intervened as soon as possible. Special considerations for complicated CHD should be considered during ECMO management.
